# Mycosynthesis of Silica Nanoparticles Using *Aspergillus niger*: Control of *Alternaria solani* Causing Early Blight Disease, Induction of Innate Immunity and Reducing of Oxidative Stress in Eggplant

**DOI:** 10.3390/antiox11122323

**Published:** 2022-11-24

**Authors:** Marzough A. Albalawi, Amer M. Abdelaziz, Mohamed S. Attia, Ebrahim Saied, Hussein H. Elganzory, Amr H. Hashem

**Affiliations:** 1 Department of Chemistry, Alwajh College, University of Tabuk, Tabuk 71491, Saudi Arabia; 2 Botany and Microbiology Department, Faculty of Science, Al-Azhar University, Nasr City, Cairo 11884, Egypt; 3 Department of Chemistry, College of Science, Qassim University, Buraidah 51452, Saudi Arabia

**Keywords:** oxidative stresses, antifungal activity, antioxidant enzymes, *Alternaria*

## Abstract

The threats to the life and production of crops are exacerbated by climate change and the misuse of chemical pesticides. This study was designed to evaluate the effectiveness of biosynthesized silica nanoparticles (SiO_2_-NPs) as an alternative to pesticides against early blight disease of eggplant. Antifungal activity, disease index, photosynthetic pigments, osmolytes, oxidative stress, antioxidant enzymes activities were tested for potential tolerance of eggplant infected with *Alternaria solani*. Silica nanoparticles were successfully biosynthesized using *Aspergillus niger* through green and ecofriendly method. Results revealed that SiO_2_-NPs exhibited promising antifungal activity against *A. solani* where MIC was 62.5 µg/mL, and inhibition growth at concentration 1000 µg/mL recorded 87.8%. The disease Index (DI) as a result of infection with *A. solani* reached 82.5%, and as a result, a severe decrease in stem and root length and number of leaves occurred, which led to a sharp decrease in the photosynthetic pigments. However, contents of free proline, total phenol and antioxidant enzymes activity were increased in infected plants. On the other hand, the treatment with SiO_2_-NPs 100 ppm led to a great reduction in the disease Index (DI) by 25% and a high protection rate by 69.69%. A clear improvement in growth characteristics and a high content of chlorophyll and total carotenoids was also observed in the plants as a result of treatment with silica nanoparticles in (healthy and infected) plants. Interestingly, the noticeable rise in the content of infected and healthy plants of proline and phenols and an increase in the activity of super oxide dismutase (SOD) and polyphenol oxidase (PPO). It could be suggested that foliar application of SiO_2_-NPs especially 100 ppm could be commercially used as antifungal and strong inducer of plant physiological immunity against early blight disease.

## 1. Introduction

The risk of plant diseases is exacerbated by the increase in the severity of climate fluctuations, which increases the risks of plant production. The eggplant is one of the most important Egyptian vegetables that is exposed to many biological stresses such as fungal diseases, nematodes and bacteria [1,2]. Soil-born fungi including *Fusarium* and *Alternaria* caused highly noticeable destructive effects on plants in organic and conventional agriculture [3,4,5]. Early blight fungal disease in eggplant that caused by *Alternaria solani* is one of the most important vegetative diseases, which results in a weakness in the quantity and quality of production [6]. As the disease causes the leaves to fall and dry, the fruits become exposed to direct sunlight and the infection of the fruits leads to rotting, as well as to their unsuitability for marketing and storage [7,8]. Symptoms appear on adult leaves in the form of circular or polygonal spots defined with clear edges and within which are characteristic typical circles [9]. All plants possess a defense system capable of resisting pathogens. The plant’s synthetic or chemical immunity can be induced by bio stimulants [10,11,12]. Mineral nutrients such as silicon or its derivatives are considered one of the most important factors for inducing plant resistance to their direct entry in strengthening the internal structures of plant tissues [13,14]. Silica is important for plant health in general, and the use of silica can be applied at any stage of the plant life cycle [15]. Silica helps in strengthening plants by establishment cell walls from the inside and these results in stronger branches that are resistant to breakage during carry flowers and fruits [16]. Silica helps plants reduce plant shock during pathogen exposure and incomplete infection stages [17]. Silica induces plant immunity against pathogens and insects, with regular use silica accumulating in plant cell walls, thus making it difficult for the pathogen to penetrate the plant cells [18]. It also controls the rate of transpiration in plants. As the plant increases silicon levels, it increases nutrient uptake and distribution, and increases the concentration of chlorophyll and carboxylase in leaves, thus the sick plant recovers from disease [19]. Silicon also improves the efficiency of photosynthesis, and also increases the endurance of leaves and the xylem, the high rates of transpiration caused by the disease, by increasing the deposition of silicon other silicon-containing substances in the cell walls of roots, leaves, twigs, and the main stem [20]. In crops, after absorbing silicon, the hardness of the cell walls improved, the cortex layer increased, thus improving the defense capacity against insects, fungi, especially blight, rot, and bacterial diseases and reducing the incidence of red and white spiders [21,22,23]. The fabrication of nanomaterials is one of nanotechnology’s most challenging and rapidly expanding sectors [24]. Nanoparticle synthesis can be accomplished through physical, chemical, or biological methods [25,26,27,28,29]. The employment of biological mass, such as bacteria, fungus, yeast, plant extract or biomass, and algal extract or biomass, is an alternative to conventional methods for the ecofriendly, healthier, faster, and less expensive synthesis of nanoparticles [30,31,32,33]. Fungi also are able to solubilize silica by producing organic acids and different complexing agents which then helps in the release of soluble silica. (i) Production of Exopolysaccharides, Hydrogen Sulphide and Siderophores. Siderophores which are chelators with a small molecular weight having a high affinity for divalent and trivalent metals. Siderophores are also thought to have a role in silicate solubilization in acidic media [34,35]. (ii) Enzyme-based mechanism. Role of enzymes in silica extraction largely lacks experimental proof. However, there are few studies which indicate that enzymes are involved in this process. Piela and co-workers proposed enzyme-based mechanism for fungus-mediated bioleaching of silica nanoparticles. Under stressed circumstances, when fungal cells come in contact with the substrate, they produce specific proteins and enzymes which interact with the silica structure of biomass leading to the formation of an enzyme-silicic acid complex. This complex is further degraded by the hydrolytic enzymes secreted by fungal cells, leading to the release of siliceous groups present in the substrate in form of silica nanoparticles [36]. Generally, filamentous fungi have played a vital role in the industrial production of biological products and in the fermentation industry due to their ability to secrete proteins and enzymes, high growth rates, ease of handling in large-scale production, and low-cost requirements for production in comparison to other microorganisms [37]. The output of the filamentous fungi cultivation is a high-quality biomass (high protein and fat levels) that can be used as an alternative added to the main diet instead of more expensive sources such as soybean and fish [38]. SiO_2_-NPs have a variety of effects on plants and are excellent in reducing agricultural pests. Silica nanoparticles have additional uses as nanopesticides, nanoherbicides, and nanofertilizers. They might also be used in plants to transport molecules like proteins and nucleotides [39]. SiO_2_-NPs are gaining a lot of interest in the agriculture sector due to their enormous surface area and small size. Herein, this study aimed to biosynthesize silica nanoparticles using *Aspergillus niger* through an ecofriendly method. Furthermore, to apply SiO_2_-NPs for biocontrol of *Alternaria solani* causing early blight disease.

## 2. Experimental Procedure

### 2.1. Chemical Used

All the chemicals, reagents, Potato dextrose agar (PDA) and Potato dextrose broth (PDB) media, acetone, sulfosalicylic acid, ninhydrin acid, glacial acetic acid, ethanol, Folin’s reagent, toluene, sodium carbonate, and sodium silicate were purchased from Sigma-Aldrich. Sodium silicate was used as a precursor. All the biological syntheses in this study were achieved using distilled water.

### 2.2. Fungi Mediated Biosynthesis of SiO_2_-NPs

Silica nanoparticles were mycosynthesized using a biomass filtrate of *A. niger* AH1, which was previously isolated, morphologically and genetically identified, and deposited in a gene bank with accession number MW680847 [33]. Two disks (1.0 cm) of *A. niger* were used to inoculate in 100 mL of PDB medium at pH 5. The mixture was then cultured for seven days at 28 ± 2 °C, and shake at 150 rpm. To obtain the extracellular components of fungal strains, after fermentation, the biomass was separated by filtration and then washed with distilled water to remove any media components. Then, approximately 15 g of fungal strain was resuspended in 100 mL distilled water for 48 h in a dark and shaking condition [40]. After filtration for the previous mixture, the biomass filtrate (supernatant) was extracted and employed in the subsequent step to create SiO_2_-NPs in the following process: 99 mL of filtrate was mixed with one ml 4 M of sodium silicate solution to prepare mixture soln. of 40 mM concentration, and followed by incubation at 25 °C overnight. The resulting turbid-white precipitate of silica nanoparticles was collected, cleaned up by washing it three times with distilled water, and then dried for four hours at 150 °C. The biomass filtrate without any metal precursor, as well as sodium silicate solution, were run as controls for the experiment.

### 2.3. Characterization of Biosynthesized Silica Nanoparticles

UV-Vis spectroscopy (JENWAY 6305 Spectrophotometer) at a wavelength of 250–500 nm was used to observe the color change brought on by the synthesis of NPs. By adopting the drop coating technique, the morphological properties (size and shape) of biosynthesized SiO_2_-NPs were examined by TEM (JEM-1230, Japan, Akishima, Tokyo 196-8558). Applying FT-IR spectroscopy (Agilent system Cary 660 FT-IR model), the functional groups’ contributions to the reducing, capping, and stabilization of SiO_2_-NPs were evaluated. The samples were analyzed for FT-IR spectra in the range of 400–4000 cm^−1^ [41]. Furthermore, the crystallinity of biosynthesized SiO_2_-NPs was investigated using X-ray diffractometer X′ Pert Pro (Philips, Eindhoven, the Netherlands). The 2θ values were in the range of 4°–80°. The corresponding voltage and current were 40 kV and 30 mA, respectively. The average size of SiO_2_-NPs synthesized by fungal metabolites was calculated using the Debye–Scherrer Equation (1) [42]:

D = (Kλ/β Cos θ)(1)

where D is the mean particle size, where K is the Scherrer constant, λ is wave length of the X-ray beam used (1.54, 184 Å), β is the Full width at half maximum (FWHM) of the peak and θ is the Bragg angle, respectively. The particle size distribution of the SiO_2_-NPs was evaluated using dynamic light scattering (DLS) measurements, which gives the hydrodynamic diameter of particles [43]. This technique was also used (Malvern Instruments Ltd., Worcestershire, UK) and carried out at the Tabbin institute for metallurgical studies, Cairo, Egypt.

### 2.4. Source of the Alternaria solani

*Alternaria solani* was brought from the plant pathology lab of botany and microbiology Dep., Faculty of science, Al-Azhar University Cairo, Egypt. The fungus was confirmed by Koch’s postulate and the suspensions of conidial were prepared and spore density was counted by a hemocytometer and adjusted to 10^6^ spores/mL [6].

### 2.5. In Vitro Antifungal Activity

Antifungal activity of biosynthesized silica nanoparticles was carried out using well diffusion method. *A. solani* was inoculated on PDA broth medium, and then incubated at 28 ± 2 °C for 3–5 days. Fungal inoculum of *A. solani* was spread on the surface of PDA plates. Then, wells with 8 mm diameter were made using sterile cork-borer on each agar plate (90 mm). The wells were filled with 100 µL of different concentrations of SiO_2_-NPs (7.81–1000 µg/mL) individually with three replicates. The culture plates were incubated at 25 °C for 7 days and the zones of inhibition were observed and measured. Radial growth of *A. solani* was evaluated at different concentrations of SiO_2_-NPs (7.81, 15.62, 31.25, 62.5, 125, 250, 500 and 1000 µg/mL). Inhibition percentage of pathogen growth was calculated using the following Equation (2):

(2) $$\mathrm{Inhibition}\;\mathrm{of}\;\mathrm{pathogen}\;\mathrm{growth}=\frac{\mathrm{Control}\;\mathrm{growth}-\mathrm{treatment}\;\mathrm{growth}}{\mathrm{control}\;\mathrm{growth}}\times 100$$

### 2.6. In Vivo Experiment

Identical and uniform 3-week-old eggplant seedlings were selected from the Agricultural Research Center (ARC), Giza, Egypt. One plant/pot (30 cm in diameter containing a mixture of sand and clay (1:3 W/W) with a total of 7 kg was planted in the Research Garden, Faculty of Science, Al-Azhar University, Cairo, Egypt. The pots were distributed with 10 replicates for each treatment. For five days, the plants were irrigated routinely. Then, before and after flowering, a one-handed pressure sprayer was used to spray treatments to the plants three times (20 mL per plant once every week (50 ppm (50 mg SiO_2_ NPs/one litter distilled water), 100 (100 mg SiO_2_ NPs/one litter distilled water) ppm of SiO_2_ NPs or sodium silicate 100 ppm (100 mg sodium silicate/one litter distilled water) separately). The aqueous solution pH was 5. The treatments were arranged as follows: T1-Healthy control, T2-Infected control, T3-healthy plants treated with SiO_2_-NPs (50 ppm), T4-healthy plants treated SiO_2_-NPs (100 ppm), T5-healthy plants treated with sodium silicate (100 ppm), T6-infected plants treated with SiO_2_-NPs (50 ppm), T7-infected plants treated with SiO_2_-NPs (100 ppm), T8-infected plants treated with sodium silicate (100 ppm). One week after transplanting the plants, an artificial infection was made with the pathogenic fungus and plants were sprayed with treatments after the week of infection (SiO_2_-NPs and sodium silicate) to assess plant resistance, record disease symptoms, as well as take samples for biochemical tests from plant samples 45 days after sowing, and the disease was examined.

Disease symptoms were recorded 45 days after transplanting, and disease severity and protection ratio were calculated, as described in Attia et al. [44] using five score classes: 0 (no symptoms), 1 (slight yellow of leaves), 2 (moderate yellow plant), 3 (wilted plant and browning of vascular bands), and 4 (plants completely destroyed). (PDI) was calculated by the equation PDI = (1n_1_+ 2n_2_ + 3n_3_ + 4n_4_) × 100/4n_t_ where n_1_–n_4_ are the number of plants in each class and N_t_ is the total number of plants. Percent protection was calculated by: Protection% = A − B/A × 100% where A = PDI in infected control plants B = PDI in infected-treated plants.

### 2.7. Measurement of Growth Traits

The samples were collected for different growth traits (shoot length, root length and number of leaves) and biochemical analysis after four weeks from transplanting.

### 2.8. Measurement of Photosynthetic Pigments

Measurement of photosynthetic pigments was carried out using the established method Vernon and Selly, [45] to assess the presence of chlorophyll a (Chl a), chlorophyll b (Chl b) and carotenoids in fresh leaves. Photosynthetic pigments were extracted from fresh leaves (1 g) using 100 mL of 80% acetone, then the color was determined spectrophotometrically at 665, 649, and 470 nm after the extract was filtered.

### 2.9. Determination of the Content of Proline

The proline content of fresh plant leaves was measured according to Bates et al. [46]. The dried shoots (1 g) were digested by 20 mL (3%) of sulfosalicylic acid. In a boiling water bath, 2 mL of the plant filtrate was mixed with 2 mL of ninhydrin acid and glacial acetic acid for one hour. The mixture was placed in an ice bath to stop the reaction. A total of 4 mL of toluene was added to the mixture, then the absorbance determined at 520 nm.

### 2.10. Determination of Total Phenol Contents

The total dry phenol content was measured using the procedure of Dai et al. [47]. One g of dried eggplant shoots was extracted in 5–10 mL of 80% ethanol for 24 h. the remaining residue was extracted three times 5–10 mL of 80% ethanol. The extract was then filled to a capacity of 50 mL with 80% ethanol, and then 0.5 mL of the extract was mixed well with 0.5 mL of Folin’s reagent and shaken for 3 min. A total of 3 mL of distilled water and 1 mL of saturated sodium carbonate solution were added and mixed. The color was detected at 725 nm after 1 h.

### 2.11. Antioxidant Enzyme Activity Assay

Superoxide dismutase (SOD) activity was evaluated according to that method described by Bergmeyer [48]. PPO activity was calculated by the procedure used by Lavid et al. [49]. SOD and PPO activities were evaluated in fresh eggplant leaves. A total of 1 g of the fresh plant was homogenized with 10 mL of phosphate buffer pH 6.8 (0.1 M), then centrifuged at 2 °C for 20 min at 20,000× *g* rpm. For SOD activity 3.6 mL of distilled water, 0.1 mL of enzyme, 5.5 mL of 50 mM phosphate buffer (pH 7.8) and 0.8 mL of 3 mM pyrogallol (dissolved in 10 mM HCl) were used. The rate of pyrogallol reduction was measured at 325 nm with UV-spectrophotometer (Jenway, Missouri City, TX, USA). For PPO activity assayed by using 125 µmol of phosphate buffer (pH 6.8), 100 µmol pyrogallol, and 0.1 mL of enzyme extract. After 5 min at 25 °C, the reaction was stopped by adding 1 mL 5% H_2_SO_4_. The developed color was determined at 430 nm.

### 2.12. Statistical Analysis

Analyses were conducted using one-way variance (ANOVA). The statistically significant differences between treatments was assessed with a *p*-value of 0.05 or lower, and the LSD test was used with CoStat (CoHort, Monterey, CA, USA) [50].

## 3. Results and Discussion

### 3.1. Biosynthesis of SiO_2_-NPs Using Biomass Filtrate of A. niger

*A. niger* strain was employed in this work as a biocatalyst for the ecofriendly production of silica nanoparticles. After being combined with a precursor and the fungal biomass filtrate, the color was changed to turbid-white as a result of the silicananoparticle synthesis, which was then calcinated at 150 °C to form SiO_2_-NPs powder. Biocatalyst exhibiting desired metabolic activity is the key step of process evaluation and decides on its final outcome. In case of presented research, biocatalysts belonging to *Fusarium* genus seemed to be ideal candidates for nano-silica biogeneration process. Among them, a number of species are plant pathogens, causing diseases of arable crops, i.e., corn, for which covers have been used in described experiments as a substrate for biotransformation. It was reasonable to assume that fungal cells can exhibit proper metabolic capacity leading to Silica-nanoparticle synthesis, in particular, since the genus *Fusarium* is known from its high activity towards production of silica nanoparticles from rice husk ash aqueous anionic complexes SiF_6_^2−^, sand and aqueous solutions of potassium silicofluoride [36]. The activities of proteins, enzymes, and carbohydrates produced by fungal metabolites may be responsible for conversion of sodium silicate to SiO_2_-NPs [51]. Kannan et al. [52] successfully fabricated SiO_2_-NPs by using *Fusarium oxysporum* and potassium silicofluoride as a precursor. Moreover, the filtrate containing the extracellular proteins secreted by the fungus *F. oxysporum* was reacted with 10^−3^ M K_2_SiF_6_ under ambient conditions for 24 h as reported by [53] for biogenic of silica nanoparticles. The results of our manuscript are agreeing with the result reported by Zamani, Jafari, Mousavi and Darezereshki [51] which uses the similar material sodium silicate to biosynthesize silica nanoparticles by using of *Saccharomyces cervisiae*. Moreover, he studied the effect of pH on sodium silicate without biomass filtrate and study the effect of pH on biomass filtrate with precursor (sodium silicate). He performed in the pH range between 6 and 11 so the effect of pH alteration in the absence of the yeast should be obtained. So, a test is designed that the pH increases in presence of the precursor while it is on the stirrer. It is observed that the pH increases from 6.51 to 10.5 has no effect on the solution and not any sediment was observed. Therefore, it can be concluded that the increase in pH does not have an effect on the production of silica nanoparticles in the absence of other parameters. This test results show that the yeast is reacted with the precursor and the pH has been affected by this reaction. A process of pH effect repeated with the yeast culture media. It is observed that also the presence of the culture medium did not yield nanoparticle production, and as a result, yeast as the precursor is the most important factor in the production of silica nanoparticles. As is clear, nanosilica production will not occur in the absence of precursor and it is observed with consideration of pH and medium effect that the presence of medium is the crucial part in the synthesis process too. This test was studied to select the best pH for biosynthesis in the range between 4–11 on the precursor only and the precursor with biomass filtrate of *Aspergillus* sp., but only the best conditions are written in the manuscript.

(3) $${\mathrm{Na}}_{2}{\mathrm{SiO}}_{3}+{\;H}_{2}O\overset{\mathrm{Yeast}\;\mathrm{Extract}}{\to }{\mathrm{SiO}}_{2}+2\mathrm{NaOH}$$

Additionally, a study was conducted by Singh, et al. [54] in which they produced silica nanospheres of the size ranging from 5 to 250 nm using liquid bacterial cultures of *Actinobacter* sp. using potassium fluorosilicate (K_2_SiF_6_) as silica precursor. They reported that synthesis of silica nanoparticles was mediated by oxidase and reductase enzymes produced by the bacteria. Addition of precursor molecules to *Actinobacter* sp. leads to ionic stress and in a way to reduce the stress these species synthesize hydrolyzing enzymes to convert the metal precursor into nanocomposites.

### 3.2. Characterization of Biosynthesized SiO_2_-NPs

#### 3.2.1. UV-Vis Spectroscopy

**Figure 1:**
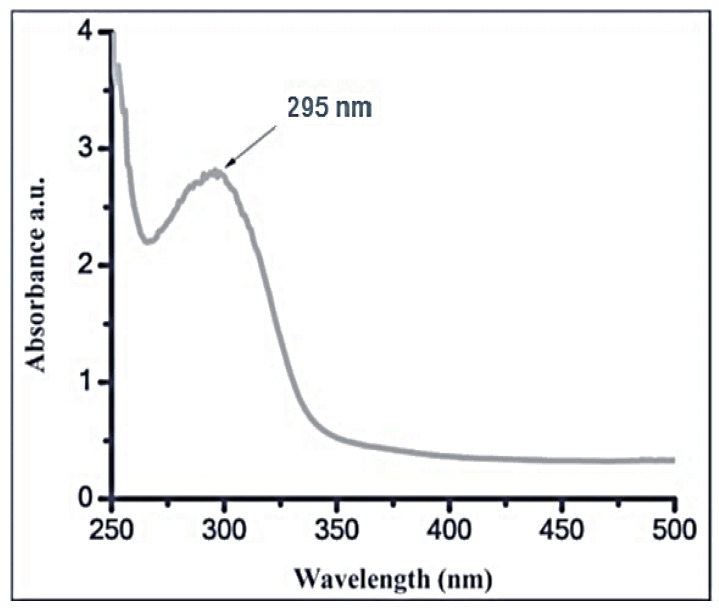
UV–vis spectrum of silica nanoparticles.

At a wavelength between 250 and 500 nm, UV-Vis spectroscopy was used to monitor the color change. The generated shape, size, and distribution of silica nanoparticles are largely affected by surface plasmon resonance (SPR). The biosynthesized SiO_2_-NPs had a single SPR band that was detected at a wavelength of 295 nm, as illustrated in Figure 1. According to Biradar et al. [55], silica nanoparticle production is indicated at absorption in the UV region at 297 nm. Similarly, Kannan et al. [52] reported that SiO_2_-NPs were successfully made utilizing *Fusarium oxysporum* and the silica surface plasmon band was found to form at about 280 nm. El-Gazzar et al. [56] found that the creation of SiO_2_-NPs occurred at around 336 nm of absorption, when exposed to a UV-visible spectral scan and corresponds to surface Plasmon resonance. A wide SiO_2_-NPs absorption band between 270 and 380 nm was observed by Ghosh et al. [57]. This indicated that cuminaldehyde had been successfully loaded into the MSNs.

#### 3.2.2. Fourier Transform Infrared (FT-IR) Spectroscopy

**Figure 2:**
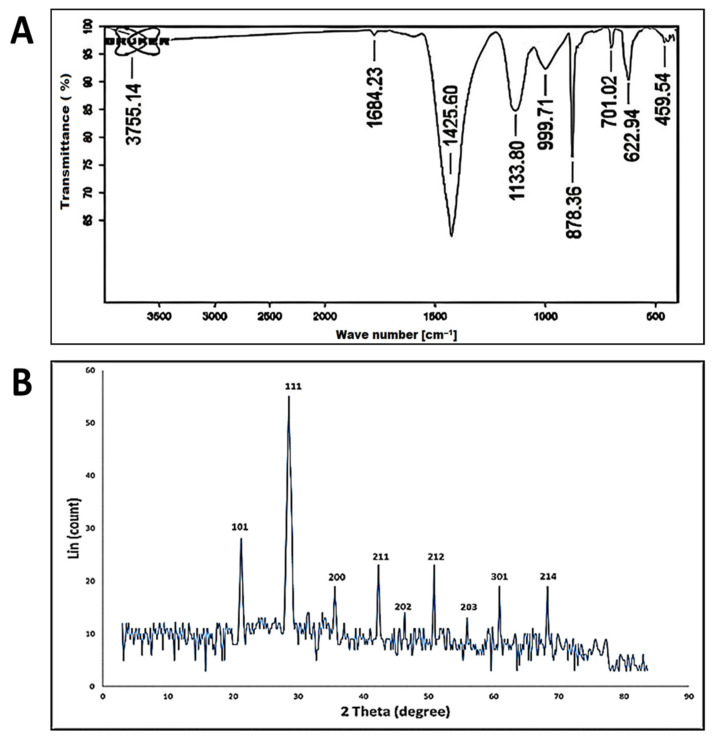
FT-IR spectra (**A**) and XRD diffractogram of SiO_2_-NPs (**B**).

Figure 2A shows the FT-IR spectra of the biosynthesized silica nanoparticles, which are analyzed in the range between 400 to 4000 cm^−1^. The absorption peaks centered at 3755, 1684, 1425, 1133, 999, 878, 701, 622 and 459 cm^−1^. The band observed at 3755 is assigned to O–H stretching vibrations and the bending vibration of C–OH [58]. The peak observed at 1684 cm^−1^ was attributed to the O–H stretching band of the surface of silanol groups [59]. The strong band observed at 1425 cm^−1^ represents the stretching mode of CO of carboxylate salt and the adsorbed CO_2_ and CO_3_^2-^ at the surface of SiO_2_-NPs [60]. The adsorption of these new functional groups on the SiO_2_-NPs surface plays a significant role in catalytic reactions [61]. The absorption peak at 1133 cm^−1^ is assigned to the Si-O-Si asymmetric stretching vibration, and the peaks at 999 cm^−1^ are attributed to the asymmetric bending and stretching vibration of Si-OH, respectively. On the other hand, the symmetric stretching vibrations of Si-O-Si appear at 878 cm^−1^ [55,62]. The observed band at 459–701 cm^−1^ indicates the successful formation of SiO_2_ [63].

#### 3.2.3. X-ray Diffraction (XRD) Pattern

The crystal structure of the as-formed SiO_2_-NPs was analyzed by XRD technique. The XRD analyzed silica nanoparticle spectra are depicted in Figure 2B. The results show that the SiO_2_-NPs have intensities at different 2θ, indicating that the silica nanoparticle structure is crystalline. The XRD pattern showed the typical Bragg reflections at 2ϴ equal to 21.2°, 28.6°, 35.6°, 42.3°, 46.36°, 50.91°, 55.94°, 60.92°, and 68.28°, which match the XRD pattern of the indices (101), (111), (200), (211), (202), (212), (203), (301) and (214), respectively. From the XRD pattern, it is confirmed that the material formed has a crystal structure. The XRD pattern observed was in accordance with the synthesis of silica nanoparticle produced by [61]. The Debye–Scherrer equation estimates that SiO_2_-NPs had an average crystallite size of 77 nm, where Peak Position (2θ) equals 55.94 ^o^ and FWHM (2θ) equals 0.1217.

#### 3.2.4. Transmission Electron Microscopy (TEM)

**Figure 3:**
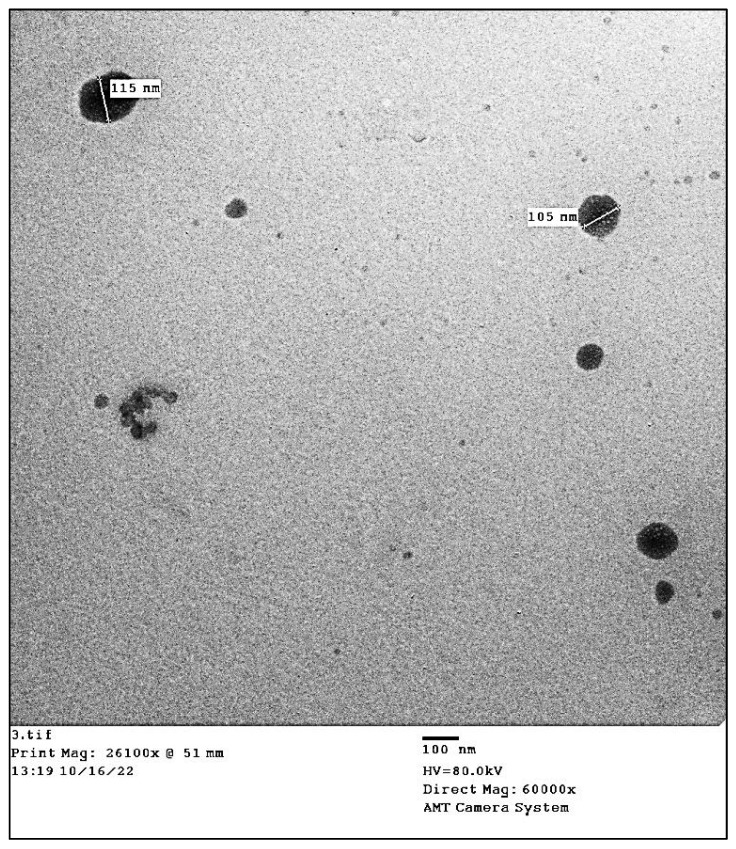
TEM image for SiO_2_-NPs showing spherical shapes.

According to Wang and Lu, [64], the activity of NPs is often associated with their shape and size, with activity progressively increasing as size decreases. Therefore, determining the size and shape of the biosynthesized SiO_2_-NPs is important. The effectiveness of the metabolites present in the fungal biomass filtrate in stabilizing the shape of silica nanoparticles is seen in Figure 3. According to the data analysis, the spherical shapes are created with diameters of between 75 and 115 nm. Vetchinkina et al. [65] reported that when *A. bisporus* humus fungi were grown with Na_2_SiO_3_, the biogenic nanoparticles had a diameter of 30–100 nm and with *A. arvensis*, the particle diameter was up to 250 nm. Additionally, *Saccharomyces cervisiae* was utilized by Zamani et al. [51] for the successful biosynthesis of spherical silica nanoparticles with a diameter size range of 40 to 70 nm. Moreover, Zielonka et al. [51] demonstrated that an *A. parasiticus* strain was able to transform the amorphous silica conglomerates into structured nanoparticles (NPs) in the process of RH biotransformation, which produces silica NPs with sizes ranging from 3 to 400 nm. The presence of biomolecules and other metabolites in biomass filtrate, which are utilized in the bio-reduction and bio-capping of produced nanoparticles, may be the cause of the variation in nano-size and nano-shape. The largest challenges that require urgent and extended studies are thought to be the behaviors of metabolites like proteins and enzymes in creating and modifying certain NPs shapes like spherical, cubic, hexagonal, nano-flowering, rectangular, and rods [40].

#### 3.2.5. Particle Size Distribution DLS

**Figure 4:**
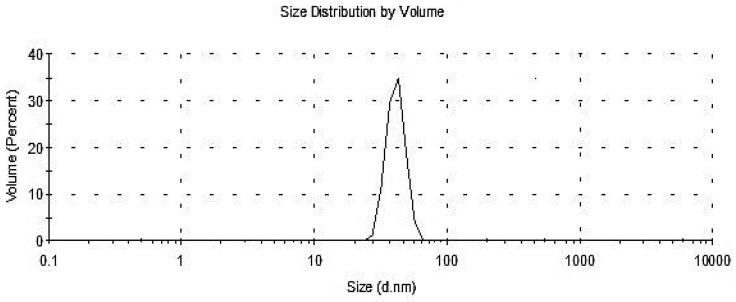
DLS analysis of biosynthesized SiO_2_-NPs colloidal solution.

Particle size distribution was analyzed by DLS and the histogram showed an average particle size (based on intensity distribution) of about 70.1 nm with a volume of 33% (Figure 4). According to El-Gazzar et al. [56], particle size distribution analyzed by DLS showed an average particle size (based on intensity distribution) of about 89 nm. Moreover, Foroutan et al. [61] reported that silica nanoparticles have a particle morphology with an average particle size of about 30–40 nm. The DLS analysis offers additional details regarding the homogeneity of particles in the colloidal solution (PDI) [66]. The solution expands when the PDI value goes up over 1.0. In this investigation, the biosynthesized SiO_2_-NPs colloidal solution exhibited a PDI value of 0.03867, indicating that the solution was homogeneous.

### 3.3. In Vitro Antifungal Activity of Silica Nanoparticles against A. solani

**Figure 5:**
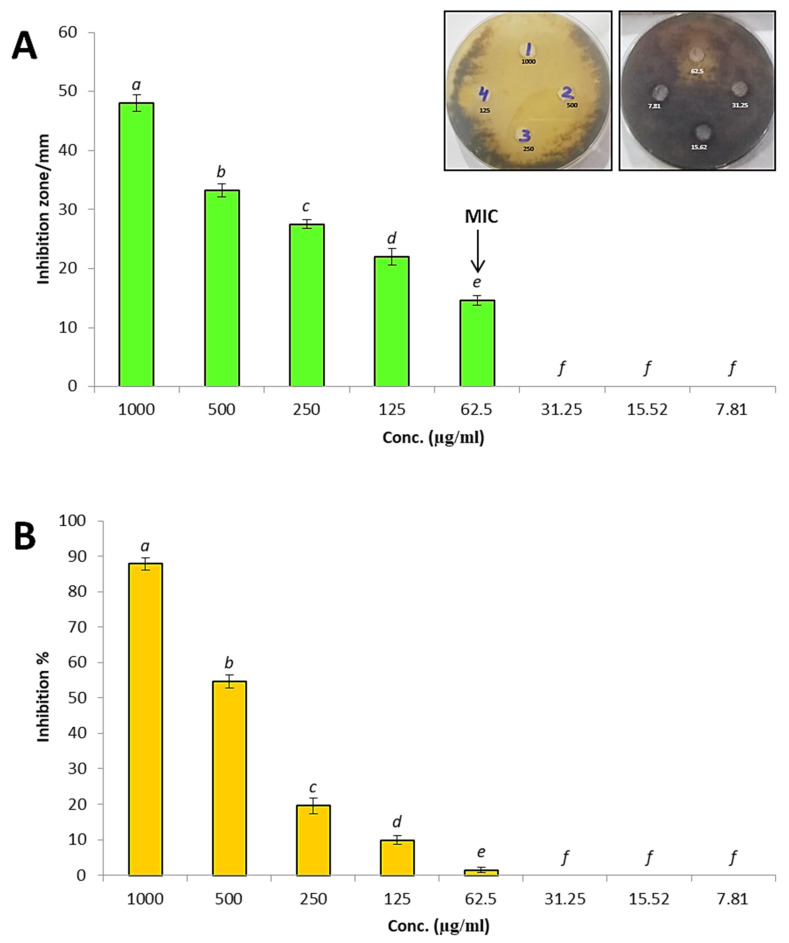
Antifungal activity of SiO_2_-NPs at different concentrations against *A. solani* (**A**,**B**): (**A**) Inhibition zones. (**B**) Inhibition percentages. (Data represent mean ± SD, n = 3). (a > b > c > d > e > f revealed to significance power). Numbers 1,2,3,4 in the upper petri dish mean 1000, 500, 250 and 125 µg/ml.

Metal nanoparticles are wildly used for biocontrol of *A. solani* causing early blight disease such as zinc, selenium, silver, copper nanoparticles [67,68,69,70]. In this study silica nanoparticles were used for biocontrol of *A. solani* causing early blight disease. Antifungal activity of SiO_2_-NPs was evaluated against *A. solani* using the agar well diffusion method, as shown in Figure 5A. Results revealed that biosynthesized SiO_2_-NPs exhibited promising antifungal activity against *A. solani* where inhibition zone 48 mm at concentration 1000 µg/mL. Also, different concentrations of silica nanoparticles were tested against *A. solani,* where results illustrated that inhibition zones were 33.2, 27.5, 22 and 14.6 mm at concentrations 500, 250, 125 and 62.5 µg/mL, respectively. On the other hand, other low concentrations 31.25, 15.62 and 7.81 µg/mL did not give any inhibition toward *A. solani*. Therefore, MIC of SiO_2_-NPs against *A. solani* was 62.5 µg/mL where this concentration exhibited minimum inhibition on growth of *A. solani.* Furthermore, the linear growth method was performed to determine inhibition percentages of SiO_2_ NPs at different concentrations toward *A. solani* as illustrated in Figure 5B. Results showed that inhibition percentages of SiO_2_-NPs at concentrations 1000, 500, 250, 125 and 62.5 µg/mL were 87.8%, 54.6%, 19.5%, 9.85% and 1.5% respectively. Meanwhile, concentrations of 31.25, 15.62 and 7.81 µg/mL did not give inhibition. Derbalah, et al. [71] fabricated silica nanoparticles to evaluate their antifungal activity against *A. solani*, and showed promising antifungal activity against *A. solani.* Ahamad and Siddiqui [72] studied effect of SiO_2_-NPs against *A. dauci* where results revealed that SiO_2_-NPs exhibited promising antifungal activity toward *A. dauci* and reduced effect of the fungus on carrot. Abdelrhim, et al. [73] reported that the silica nanoparticles significantly reduced mycelial radial growth of *Rhizoctonia solani,* and also induced innate immune response in wheat. Khan and Siddiqui [74] used SiO_2_-NPs as seed priming for controlling of *R. solani,* where results illustrated that SiO_2_-NPs exhibited antifungal activity against *R. solani,* and this led to enhance growth, chlorophyll and defense enzymes.

### 3.4. Disease Index

As shown in the Table 1, *Alternaria* infection caused a high DI, reaching 82.5%. The results of this study agree with several studies on *Alternaria* [6,44,75]. Reducing the severity of the disease and increasing the protection% considers the most important indications of the occurrence of plant resistance [76]. Biosynthesized nanoparticles are considered a magic solution to overcome the plant biotic stresses, especially fungal plant diseases, because they are very highly efficiency as bio fertilizers and have antifungal properties [77,78,79]. Silica helps plants reduce plant shock during pathogen exposure and incomplete infection stages [17]. The results of the current study showed that treatment with SiO_2_-NPs caused a decrease in the DI% and an increase in protection% compared to the untreated plants. In this regard, application of SiO_2_-NPs (either 50 or 100 ppm) on infected plants, showed a higher decrease in the disease symptoms and PDI and thus gives a high percentage of protection compared with untreated infected plants. Treatment of infected plants with SiO_2_-NPs (50 ppm) and (100 ppm) reduced DI% to 23% and 25% and high protection by 69.99% and 57.57%, respectively and next came silicon that recorded DI% 60% and protection 27.27%. Scientific reports have proven that silicon reduces the toxic effect on plants resulting from various stresses, as it works to strengthen cell walls and structural barriers, preventing the penetration of pathogens or impeding the movement of the pathogen inside the plant [80,81].

### 3.5. Measurement of Growth Traits

**Figure 6:**
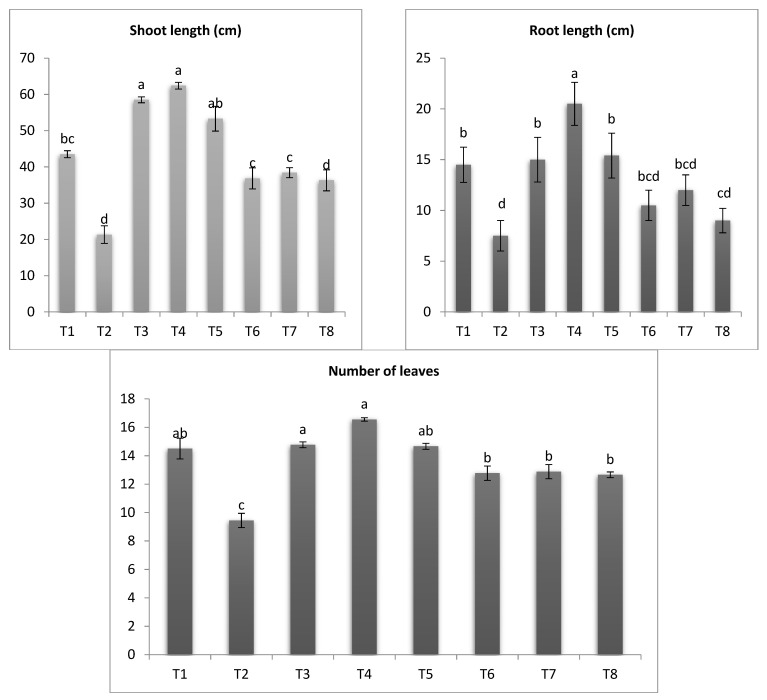
Effect of SiO_2_-NPs and sodium silicate on morphological characters of *S. melongena.* (Data represent mean ± SD, n = 3). (a–d revealed to significance letters).

Heavily recently studies demonstrated that fungal plant diseases caused significant changes in vegetative growth [82,83,84]. Results in Figure 6 shows *A. solani* affected greatly reduced eggplant growth traits; shoot length, root length and number of leaves by 50.96%, 48.27% and 34.89%. Our results are consistent with [44,85,86]; they concluded that *Alternaria* infection causes a significant decrease in vegetative traits of many crops. In this respect, this severe deficiency can be explained by dangerous disturbances within the cell metabolism as a result of oxidative blasts during the *A. solani* infection process [85,87]. On the other hand, application of SiO_2_-NPs resulted to enhancement of both healthy and infected plants compared to control. Likewise, treatment infected plants with SiO_2_-NPs (100 ppm) and (50 ppm), showed a significant increase in shoot length, root length and number of leaves and next came Si, respectively. The highest increase in shoot length, root length and number of leaves was recorded in SiO_2_-NPs 100 ppm treated plants. Interestingly, the application of SiO_2_-NPs on healthy plants showed a significant improvement in the vegetative growth characteristics as shown in the Figure 6. Many previous studies support these results [73,87,88]. Many recently studies proved that the plant resistance against biotic as well as abiotic stresses can be enhanced and stimulated by application of safe organisms and their metabolites through different mechanisms [88,89,90,91]. The results can be explained by the fact that fertilizing with silicon increases the efficiency of phosphorous uptake, which is reflected in an increase in lipid substances in plant cells, which increases the efficiency of roots to absorb. In addition, silicon supports the hardness of cell walls, which prevents the penetration of pathogenic fungi and reduces stress [39,92,93].

(T1-Healthy control, T2-Infected control, T3-healthy plants treated with SiO_2_-NPs (50 ppm), T4-healthy plants treated SiO_2_-NPs (100 ppm), T5-healthy plants treated with sodium silicate (100 ppm), T6-infected plants treated with SiO_2_-NPs (50 ppm), T7-infected plants treated with SiO_2_-NPs (100 ppm), T8- infected plants treated with sodium silicate (100 ppm)).

### 3.6. Photosynthetic Pigments

**Figure 7:**
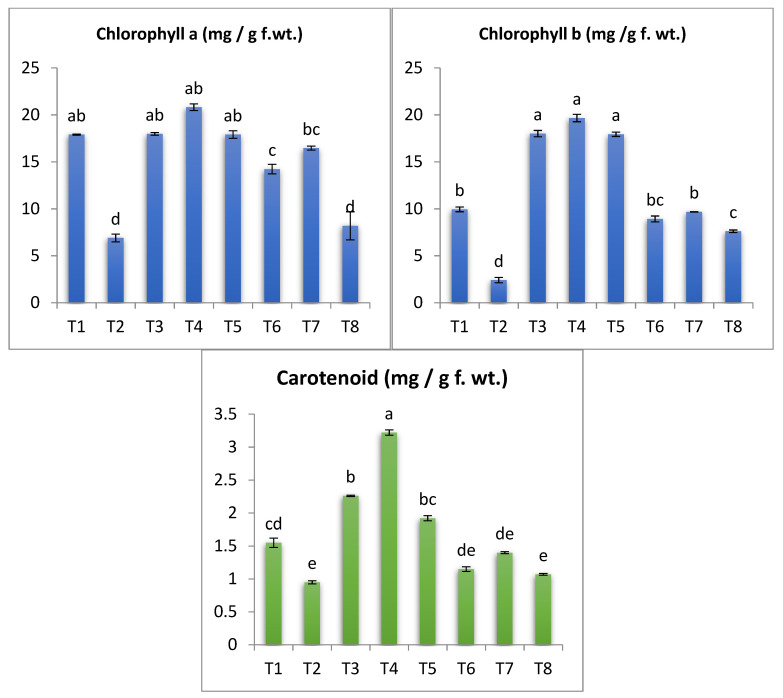
Effect of silica nanoparticles and SiO_2_ on photosynthetic pigments of *S. melongena.* (Data represent mean ± SD, n = 3). (a–e revealed to significance letters).

As shown in Figure 7, a sharp decrease in chlorophyll (a, b) and carotenoid was observed due to infection with *Alternaria* fungus compared to uninfected plants. These results are in agreement with several studies [6,46,87,94]. This acute deficiency in the components of chlorophyll and carotenoids may be due to the plant’s failure to capture light due to a deficiency in the leaf area responsible for light capture and photosynthesis, leaves and plant height, or it may also be due to oxidative explosion and increased activity of chlorophyll-degrading enzymes and chlorophyllase under the conditions of injury [6,81,88]. However, *Alternaria*-infected plants treated with silica nanoparticles (50 and 100 ppm) or Si showed a marked increase when compared with untreated infected plants. Concerning the effect SiO_2_-NPs and SiO_2_ on the infected plants with *A. solani*, it was found that SiO_2_-NPs 100 and 50 ppm indicated a greatly significant rise in the contents of Chl a and Chl b. The uninfected eggplant seedlings treated with SiO_2_-NPs or SiO_2_ presented a major increase in chlorophyll pigments a and b. Also, the obtained results illustrated that in *A. solani* infection plants, the content of carotenoids was increased in response to the treatment with SiO_2_-NPs. This increase could be attributed to improved number of leaves, stomata conductance, transpiration rate and/or cell size and number [95,96,97]. This improvement in the level of photosynthetic pigments may be explained by the fact that SiO_2_-NPs have been described as plant immune-stimulants [94,98]. It may trigger NADPH oxidase activity, thereby stimulating the production of H_2_O_2_. Thus, SiO_2_-NPs could stimulate reactive oxygen species (ROS) in plants. Silicon also increases the productivity of photosynthesis, and also rises the strength of leaves and the xylem, the high rates of transpiration caused by the disease by increasing the deposition of silicon in the cell walls of roots, leaves, twigs, and the main stem [20].

## 4. Oxidative Stress Markers

**Figure 8:**
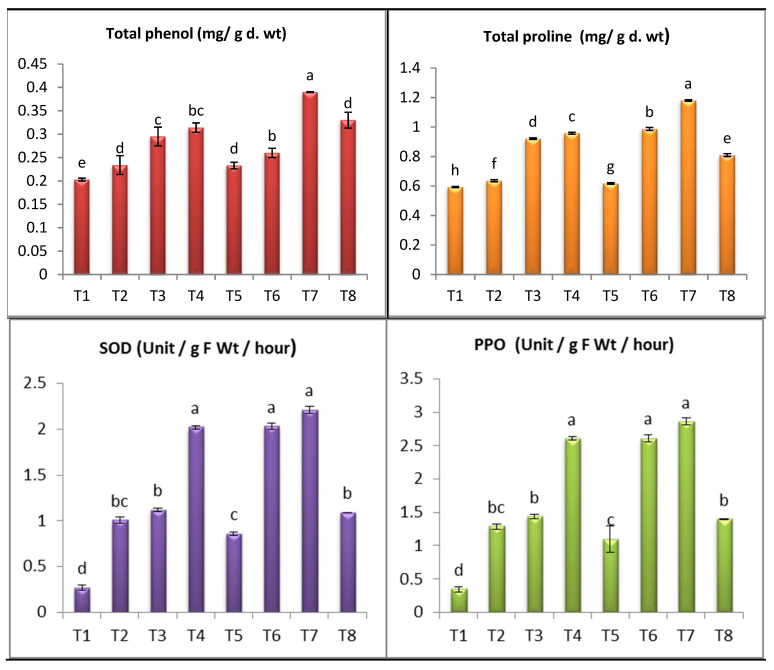
Effect of SiO_2_-NPs and SiO_2_ on oxidative stress markers of *S. melongena*. (a–h revealed to significance letters).

*Alternaria* infected eggplants showed significant increases in contents of free proline, total phenols by 7.25%, 15.27%, respectively, relative to uninfected control plants (Figure 8). Our results are in agreement with other studies [4,6,99,100]. The increase of proline and phenols acts as one of the imperative biological activities as it works a critical role in capturing free radicals, keeping cells from oxidation, and providing plant cells with energy [101,102]. Moreover, application of SiO_2_-NPs or SiO_2_ on infected plants resulted in significant increase in content of proline by 85.53%, 55.18%, and 27% at SiO_2_-NPs 100 ppm, 50 ppm and SiO_2_. Also, contents of phenols increased by 66.66%, 41.02% and 11.11% with SiO_2_-NPs 100 and 50 ppm, respectively, when compared untreated infected plants. The highest increase in contents of proline, total phenols was recorded in SiO_2_-NPs 50 ppm treated plants. Proline accumulation in eggplants shoots prevents the photosynthetic process throughout inhibiting injury of photosynthetic pigments caused by ROS [45,100,101]. Phenols perform an chief role in capturing free radicals, which decreases oxidative stress in cells [103]. The increase of phenolic compounds is an adaptive approach against disease [104]. Through our results, a significant increase in the content of proline and phenol was observed due to the addition of silica nanoparticles, especially concentration (100 ppm), which confirms the role of SiO_2_-NPs in inducing plant immunity, as the accumulation of proline in plants prevents damage to photosynthetic pigments by capturing free radicals that cause damage and failure in the photosynthesis process [105,106]. In these results, a clear and positive link to the use of SiO_2_-NPs in terms of improving vegetative growth characteristics, increasing the level of photosynthetic pigments, and raising the content of proline and phenol in infected plants cells, which supports the role of silicon in strengthening plant immunity against stress, which was confirmed by many previous studies [14,107,108].

To achieve a stronger sign on resistance, mean activities of superoxide dismutase and polyphenol oxidase of the tested eggplants were established in this experiment. SOD and PPO activities were greater in the Alternaria–infected plants and treated with (SiO_2_-NPs 100, 50 ppm and SiO_2_). The activity of SOD and PPO were increased in Alternaria-infected eggplants compared to healthy plants. The activity of SOD and PPO was boosted in infected plants comparing to healthy control plants (Figure 8). Application of (SiO_2_-NPs 100 ppm, SiO_2_-NPs 50 ppm and sodium silicate 100 ppm) increased the activity of SOD by (643.46%, 301.17% and 218.86%) and PPO by (312.39%, 642.03% and 215.64%) respectively, over healthy controsl. Moreover, application of (SiO_2_-NPs 100 ppm and SiO_2_-NPs 50 ppm) increased the activity of SOD by (119.46% and 101.58%) and PPO by (122% and 103%) respectively, over infected plants (Figure 8). Meanwhile, sodium silicate 100 ppm was the least effective and increased (SOD and PPO) activity by (7.24% and 9.01%) (Figure 8). Under non-infected conditions, application of SiO_2_-NPs 100 ppm, SiO_2_-NPs 50 ppm and sodium silicate 100 ppm boosted the activities of SOD and PPO in treated plants as compared with control. The study carried out by Kiadaliri et al. [109] reported that antioxidant enzymes SOD and PPO provide a large number of defensive enzymes associated with infection. These enzymes act as initial steps in increasing plant resistance to various stresses as well as the formation of phenolic compounds. The plant presented different mechanisms to cope with infection stress as they increase the activity of certain antioxidant enzymes to keep ROS at the lower level in the cell [110]. Increasing the activity of antioxidant enzymes (SOD, PPO) as a result of treatment with SiO_2_-NPs on infected plants provides strong evidence of induction of resistance against the disease. Antioxidant enzymes mitigate the oxidative blast caused by injury by capturing free radicals from cells [111].

## 5. Conclusions

In the current study, SiO_2_-NPs were successfully mycosynthesized using *A. niger*, and fully characterized using Uv-Vis, FTIR, XRD, TEM and DLS techniques. Mycosynthesized SiO_2_-NPs exhibited promising antifungal activity toward *A. solani*. The application of SiO_2_-NPs at different concentrations (50 and 100 ppm) greatly offers the possibility of early blight recovery in eggplant. For more, mycosynthesized SiO_2_-NPs can be applied as a plant growth-promoting agent instead of chemical synthesized fertilizers in healthy and infected eggplant. Additionally, SiO_2_-NPs showed the highest increase in contents of proline and phenols. It is worth mentioned that the prepared SiO_2_-NPs can modify plant antioxidant defense system by inducing overexpress oxidative stress in plants. So, the usage of the tested SiO_2_-NPs can increase not only the anti-fungal activity, but also the systematic immune response of treated plants. Therefore, mycosynthesized SiO_2_-NPs can be applied as a confident and safe alternative bio fungicide against *Alternaria solani* causing early blight disease.

## Figures and Tables

**Table 1:** Effect of SiO_2_-NPs and SiO_2_ on *A. solani* early blight disease index of *S. melongena*.

Treatment	Disease Symptoms Classes					DI (%)	Protection (%)
	0	1	2	3	4		
Control Infected	0	0	1	5	4	82.5	0
Infected + SiO_2_-NPs 50 ppm	4	0	4	2	0	35	57.57
Infected + SiO_2_-NPs 100 ppm	5	1	3	1	0	25	69.69
Infected + sodium silicate 100 ppm	2	1	1	3	3	60	27.27

## Data Availability

Data are contained within the article.
